# Ceramide kinase is required for a normal eicosanoid response and the subsequent orderly migration of fibroblasts[S]

**DOI:** 10.1194/jlr.M048207

**Published:** 2014-07

**Authors:** Dayanjan S. Wijesinghe, Matthew Brentnall, Jennifer A. Mietla, L. Alexis Hoeferlin, Robert F. Diegelmann, Lawrence H. Boise, Charles E. Chalfant

**Affiliations:** * Department of Surgery Virginia Commonwealth University-School of Medicine, Richmond, VA 23298; †† Department of Biochemistry and Molecular Biology, Virginia Commonwealth University-School of Medicine, Richmond, VA 23298; † Hunter Holmes McGuire Veterans Administration Medical Center, Richmond, VA 23249; § Hematology and Medical Oncology, Emory School of Medicine, Atlanta, GA 30322; ** Miller School of Medicine, University of Miami, Miami, FL 33136; §§ The Massey Cancer Center, Richmond, VA 23298

**Keywords:** wound healing, ceramide-1-phosphate, lipidomics

## Abstract

In these studies, the role of ceramide-1-phosphate (C1P) in the wound-healing process was investigated. Specifically, fibroblasts isolated from mice with the known anabolic enzyme for C1P, ceramide kinase (CERK), ablated (CERK^−/−^ mice) and their wild-type littermates (CERK^+/+^) were subjected to in vitro wound-healing assays. Simulation of mechanical trauma of a wound by scratching a monolayer of fibroblasts from CERK^+/+^ mice demonstrated steadily increasing levels of arachidonic acid in a time-dependent manner in stark contrast to CERK^−/−^ fibroblasts. This observed difference was reflected in scratch-induced eicosanoid levels. Similar, but somewhat less intense, changes were observed in a more complex system utilizing skin biopsies obtained from CERK-null mice. Importantly, C1P levels increased during the early stages of human wound healing correlating with the transition from the inflammatory stage to the peak of the fibroplasia stage (e.g., proliferation and migration of fibroblasts). Finally, the loss of proper eicosanoid response translated into an abnormal migration pattern for the fibroblasts isolated from CERK^−/−^. As the proper migration of fibroblasts is one of the necessary steps of wound healing, these studies demonstrate a novel requirement for the CERK-derived C1P in the proper healing response of wounds.

Healing of cutaneous wounds proceeds through four distinct, yet overlapping stages. These stages include hemostasis, inflammation, proliferation, and remodeling (1). During hemostasis, blood vessels constrict to minimize bleeding. The inflammatory stage of wound healing is then initiated and consists of vasodilation, increased vascular permeability, and neutrophil and monocyte (macro­phage) infiltration to the wound site. This stage clears bacteria and cellular debris. The proliferation phase begins toward the end of the inflammatory stage and involves the migration and proliferation of fibroblasts and keratinocytes. This stage also includes the formation of new blood vessels, induced by migrating endothelial cells, resulting in the formation of abundant granulation tissue. The final stage of wound healing is the maturation and remodeling phase. In this stage, new epithelium is formed, and the collagen content in the wound is increased and deposited in an orderly fashion. The high level of collagen cross-linking in this stage leads to a healed wound with ∼80% the strength of the original skin.

Fibroblasts are one of the most important groups of cells taking part in the wound-healing process. During the proliferation stage of wound healing, the fibroblasts in the surrounding tissue are stimulated to proliferate followed by migration into the wound. Fibroblasts provide the necessary extracellular matrix promoting the migration of additional cell types necessary for the completion of the wound-healing process. Thereafter, the fibroblasts alter their phenotype into myofibroblasts. Contraction of the myofibroblasts induces the wound edges to conjoin, minimizing the exposed surface area (1, 2). While not as predominant as in rodents, wound contraction still accounts for up to 88% of the mechanism toward wound closure in humans (1) indicating the relevance of these cells in human wound healing. Inhibition or loss of fibroblast migration leads to these cells remaining at the margins and thus causes abnormal wound healing (3). On the other hand, if there is increased, disorganized, or random migration of fibroblasts, the deposition of collagen would be uneven and unregulated, leading to hypertrophic scar formation or healed wounds with compromised strength (4–7).

Several published studies demonstrate the importance of eicosanoids, a class of inflammatory lipids, in the migration of fibroblasts (7–11). Green et al. (8) demonstrated that products of 5-lipoxygenase and cyclooxygenase (COX) are important in the regulation of wound closure in NIH/3T3 cells, while Kohyama et al. (9) demonstrated that prostacyclin analogs inhibit the migration of fibroblasts. Rieger et al. (7) demonstrated that 5- and 12-hydroxyeicosatetranoic acid (HETE) cause a dose-dependent increase in the chemotaxis of fibroblasts. Su et al. (11) demonstrated that nonsteroidal anti-inflammatory drugs result in excessive scar formation due to enhanced fibroblast migration and proliferation. These studies demonstrate an important role for eicosanoids in the migration and proliferation of fibroblasts.

Group IVA cytosolic phospholipase A_2_ (cPLA_2_α) is one of the major phospholipases activated in response to mechanical insults, which leads to the production of arachidonic acid (AA). AA is metabolized enzymatically to various eicosanoids (prostaglandins, leukotrienes, thromboxanes, etc.). Ceramide-1-phosphate (C1P) is a potent and specific activator of cPLA_2_α, and our published reports demonstrate the requirement for ceramide kinase (CERK)-derived C1P in the release of AA in response to inflammatory agonists (12–16). Here, we explore the hypothesis that CERK-derived C1P regulates the migration of fibroblasts due to its regulatory role in eicosanoid synthesis. We demonstrate that genetic loss of CERK severely affects the ability of fibroblasts to synthesize eicosanoids in response to mechanical injury as well as in wound biopsies. This compromised eicosanoid cascade resulted in the migration of fibroblasts in a random pattern. The requirement of C1P for orderly fibroblast migration correlates with the observed peak production of C1P in the fibroplasia stage of human wound healing. Collectively, these findings demonstrate that the presence of CERK-derived C1P is required for the proper eicosanoid response and migration of fibroblasts into a wound site.

## EXPERIMENTAL PROCEDURES

### Materials

DMEM, RPMI, FBS, and penicillin/streptomycin (100 U/ml penicillin G sodium and 100 μg/ml streptomycin sulfate) were obtained from Invitrogen Life Technologies (Carlsbad, CA). The HPLC used was a Shimadzu Prominence LC-20-AD system, and the mass spectrometer was a 4000 QTRAP® from ABSciex. Prior to mass spectrometric analysis, lipids were separated by reverse-phase chromatography using a Phenomenex Kinetex 2.6 μm C18 100A 50 × 2.1 mm reverse-phase HPLC column (Torrance, CA). HPLC-grade methanol, HPLC-grade chloroform, and American Chemical Society-grade formic acid (EMD Chemicals) were purchased from VWR (Bridgeport, NJ).

### Isolation of mouse embryonic fibroblasts

Primary mouse embryonic fibroblasts (MEFs) were isolated from 13- or 14-day pregnant wild-type (CERK^+/+^) or knockout (CERK^−/−^) females in BALB/c genetic background as described previously (16). The harvested MEFs were cultured in high glucose DMEM (Invitrogen) supplemented with 20% FBS (Invitrogen) and 2% penicillin/streptomycin (BioWhittaker) at standard incubation conditions. These cells were either used at the primary stage or passaged every 3 days for 20 serial passages to obtain immortalized MEFs.

### Scratch-induced mechanical trauma of fibroblasts

Primary or immortalized MEFs (2 × 10^6^) obtained from CERK-null mice and their wild-type littermates were plated on 100 mm tissue culture plates in DMEM supplemented with 10% FBS and 2% penicillin/streptomycin. The cells were allowed to adhere overnight under standard incubation conditions. Following overnight incubation, the medium was changed from full serum to 2% serum, and a single scratch was made on the monolayer along the diameter of the plate with a 200 μl pipette tip. The migration of the fibroblasts into the cleared area was monitored via video microscopy.

### Steady-state AA labeling of fibroblasts

Primary or immortalized MEFs (2 × 10^6^) obtained from CERK-null mice and their wild-type littermates were plated on 100 mm tissue culture plates in DMEM supplemented with 10% FBS and 2% penicillin/streptomycin and labeled with 0.25 μCi/ml ^3^H AA as previously described (17). The resultant monolayer obtained the next morning was rested for 2 h in medium containing 2% FBS and 2% penicillin/streptomycin. The monolayer was then subjected to a scratch with a 200 μl pipette tip. Medium was collected at the indicated time points, and liberated AA was measured via scintillation.

### Sphingolipid and eicosanoid analysis

For sphingolipid extraction, cells (1 × 10^6^) were harvested using a modified Bligh-Dyer protocol. Briefly, the plates were placed on ice, and the medium was transferred to another tube and used in the quantitative and qualitative analysis of eicosanoids as detailed subsequently. For sphingolipid analysis, cells were washed twice with ice-cold PBS and harvested by scraping in 200 μl of PBS followed by sonication to obtain a homogenous mixture. Lipids were extracted from the remaining cells using a modified Bligh and Dyer method and analyzed as described by Wijesinghe et al. (18). Briefly, to 200 μl of the cells in PBS, 1.5 ml of 2:1 methanol-chloroform was added. The samples were “spiked” with 500 pmol of d_18:1/12:0_ C1P, sphingomyelin, ceramide, and monohexosylceramide as the internal standard (Avanti). The mixture was sonicated to disperse the cell clumps and incubated for 6 h at 48°C. Following incubation, the extracts were transferred to a new glass tube, dried down, and reconstituted in methanol (600 μl) by sonicating and incubating at 48°C for 15 min. The reconstitution in methanol and incubating at 48°C is a new addition to our previously published method (18) and was incorporated into the existing method to ensure proper solubilization of the long-chain sphingolipids. The lipid extract thus obtained contained insignificant levels of proteins as measured by the Bradford assay (data not shown) and was used in the analysis of the sphingolipids C1P, ceramide, sphingomyelin, and monohexosylceramide. The lipids were separated using a Kinetix C18 column (50 × 2.1 mm, 2.6 µm; Phenomenex) on a Prominence HPLC system (Shimadzu) and eluted using a linear gradient (solvent A, 58:41:1 CH_3_OH/water/HCOOH 5 mm ammonium formate; solvent B, 99:1 CH_3_OH/HCOOH 5 mm ammonium formate, 20–100% B in 3.5 min and at 100% B for 4.5 min at a flow rate of 0.4 ml/min at 60°C). ESI-MS/MS using an AB Sciex 4000 QTRAP® instrument (Applied Biosystems, MDS Sciex) was used to detect C1P, ceramide, sphingomyelin, and monohexosylceramide under positive ionization.

Eicosanoids were analyzed from culture medium as we described previously (19–20). Briefly, to 4 ml of medium, 10% methanol (400 μl) and glacial acetic acid (20 μl) were added. The samples were spiked with internal standard mixture (100 μl) containing the following deuterated eicosanoids (100 pg/μl, 10 ng total): (*d*_4_) 6k prostaglandin F_1α_ (PGF_1α_), (*d*_4_) prostaglandin F_2α_ (PGF_2α_), (*d*_4_) prostaglandin E_2_ (PGE_2_), (*d*_4_) prostaglandin D_2_ (PGD_2_), (*d*_8_) 5-HETE, (*d*_8_) 15-HETE, (*d_8_*) 14,15-epoxyeicosatrienoic acid, and (*d*_8_) AA. Strata-X SPE columns (Phenomenex) were washed with methanol (2 ml) and then distilled water (2 ml). The samples were applied to the column. Thereafter, the sample vials were rinsed with 5% methanol (2 ml), which was then used to wash the columns. Finally, the eicosanoids were eluted with isopropanol (2 ml). The eluent was dried under vacuum, and the samples were reconstituted in 50:50 ethanol-distilled water (100 μl) for LC/MS/MS analysis. The lipid extract thus obtained contained insignificant levels of proteins as measured by the Bradford assay (data not shown), and the reconstituted eicosanoids were analyzed via HPLC ESI-MS/MS. A 30 min reversed-phase LC method utilizing a Kinetex C18 column (100 × 2.1 mm, 2.6 µm) was used to separate the eicosanoids at a flow rate of 200 µl/min at 50°C. The column was equilibrated with 100% solvent A [acetonitrile-water-formic acid (40:60:0.02, v/v/v)] for 5 min, and then 10 µl of sample was injected. The 100% solvent A was used for the first minute of elution. Solvent B [acetonitrile-isopropanol (50:50, v/v)] was increased in a linear gradient to 25% solvent B to 3 min, to 45% until 11 min, to 60% until 13 min, to 75% until 18 min, and to 100% until 20 min. The 100% solvent B was held until 25 min, decreased to 0% in a linear gradient until 26 min, and then held until 30 min. The eicosanoids were then analyzed using a tandem quadrupole mass spectrometer (AB Sciex 4000 QTRAP®, Applied Biosystems) via multiple-reaction monitoring in negative-ion mode. Eicosanoids were monitored using analyte specific precursor → product multiple reaction monitoring pairs, which can be found in supplementary Table I. The mass spectrometer parameters used were as follows: curtain gas: 30; CAD: high; ion spray voltage: −3,500 V; temperature: 500°C; gas 1: 40; gas 2: 60; declustering potential, collision energy, and cell exit potential vary per transition.

### Morphological analysis of fibroblasts

Cells were grown on glass coverslips (Fisher) coated with 5 μg/cm^2^ fibronectin (Chemicon International) in a 24-well plate and then fixed for 10 min with PHEMO buffer (68 mM PIPES, 25 mM HEPES, 15 mM EGTANa_2_, 3 mM MgCl_2_6H_2_O, 10% DMSO, pH 6.8) supplemented with 3.7% formaldehyde (Fisher), 0.05% glutaraldehyde (Fisher), and 0.5% Triton X-100 (Fisher). Cells were washed with PBS and blocked for 10–15 min in 10% goat serum (Cellgro). Actin was labeled by staining with Alexa Fluor 555 phalloidin (Invitrogen) at 1:40 in PBS, and DNA was stained with 300 nM 4’,6-diamidino-2-phenylindole, dilactate (Invitrogen) for 5–10 min in distilled water. Coverslips were then mounted on microslides (Fisher) using 20–30 μl polyvinyl alcohol mounting medium with DABCO antifade (Fluka) and allowed to dry overnight at room temperature in the dark. Cell morphology was determined by visualizing cells using a point scanning laser confocal microscope (LSM 510 META).

### Migration analysis of fibroblasts

Cells were seeded into 35 mm × 10 mm cell culture dishes (Corning) and allowed to grow until confluency. Initiation of migration was achieved by scratching confluent cells with a p10 pipette tip. Motility was measured on a Ziess Axiovert 200M microscope mounted with a Perkin Elmer Ultraview ERS enclosed in a heated chamber (37°C) with 5% CO_2_ injection. Images were acquired every 10 min for at least 15 h. After image acquisition, Volocity software was used to determine percent wound closure at designated time points and to analyze single cell tracks over time. Cell tracking data included average velocity and meandering index (calculated for each track by measuring the displacement of the cell from origin at time of observation and dividing by the track length), and *t*-test was used to determine significance.

### Rescue of CERK^−/−^ fibroblasts via exogenous addition of eicosanoids

Immortalized MEFs (2 × 10^6^) obtained from CERK-null mice and their wild-type littermates were plated on 100 mm tissue culture plates in DMEM supplemented with 10% FBS and 2% penicillin/streptomycin. The cells were allowed to adhere overnight under standard incubation conditions. The resultant monolayer obtained the next morning was rested for 2 h in medium containing 2% FBS and 2% penicillin/streptomycin. The monolayer was then subjected to a scratch with a 200 μl pipette tip, and the medium was supplemented either with ethanol (vehicle) or the relevant deficient eicosanoids (0.9 ng/ml AA, 0.3 ng/ml PGE_2_, 0.5 ng/ml 6-keto PGF_1_α, 0.44 ng/ml 5-HETE, 0.5 ng/ml 11-HETE, 1.0 ng/ml 12-HETE, and 1.1 ng/ml 15-HETE). The migration of the fibroblasts into the cleared area was monitored photographically, recorded at 3 h, and the percent closure of the scratched area was calculated with the aid of ImageJ and Microsoft Excel.

### Human in vivo wound analysis

Using alcohol and Betadine, the site of implantation was sterilized and anesthetized using 3 cc lidocaine (1%) without epinephrine. Five 6.0 cm high-porosity polytetrafluoroethelene (PTFE; Custom Profile Extrusions, Tempe, AZ) tubes were implanted subcutaneously into the inner aspect of the upper arms of healthy volunteer subjects. Standardized placement was made by a 5.5 cm cannulation of the subcutaneous tissue in a proximal direction. Using a sterile 14 gauge trochar containing PTFE tubing, the skin was punctured, and the trochar was inserted subcutaneously arising through the skin 5.5–6.0 cm away. The trochar was then removed, and the proximal and distal ends of the PTFE tubing were sutured to the skin using a single 5.0 nylon suture. A punch biopsy (10 mm) was taken from near the surgical site, and the dissected dermal portion was stored in 10% formalin and used for comparison in the lipid analyses (day 0). The implantation sites and the punch biopsy sites were covered with antibiotic ointment and a transparent surgical dressing. On days 3, 5, 7, and 14, one PTFE tube was removed and stored in 10% formalin. Lipid analysis was carried out via HPLC ESI-MS/MS.

### CERK knockout mouse and animal welfare assurances

Breeding pairs for the CERK^+/+^ and CERK^−/−^ counterparts were obtained from Novartis Pharma as a gracious gift from Dr. Frederic Bornancin. All cells derived from these mice were genetically verified for each experiment as described by Bornancin and coworkers (21). Animal experiments were conducted at Virginia Commonwealth University (VCU; Richmond, VA). The research protocol Institutional Animal Care and Use Committee (IACUC) AM10089 was approved by the VCU IACUC in accordance with the US Department of Agriculture (USDA) Animal Welfare Regulations; the Public Health Service Policy on the Humane Care and Use of Laboratory Animals; and the United States Government Principles for the Utilization and Care of Vertebrate Animals Used in Testing, Research, and Training. VCU is in compliance with all provisions of the Animal Welfare Act and other federal statutes and regulations relating to animals. VCU is registered under the Animal Welfare Act as a Class “R” research facility with the USDA-Animal and Plant Health Inspection Service-Animal Care (registration number 52-R-0007). The Office of Laboratory Animal Welfare has approved the Animal Welfare Assurance for VCU in compliance with the U.S. Public Health Policy (assurance number A3281-01).

### Institutional Review Board assurance of human studies

All human studies were carried out under the approval of the Institutional Review Board (IRB) of VCU-School of Medicine (IRB number 11087) and written informed consent was obtained from all participants.

### Statistical analysis

Data are expressed as mean ± SD. A Student’s *t*-test was utilized when comparing two independent groups against each other for statistical significance. Where statistical comparison of four or more independent groups was required, a one-way ANOVA with Tukey’s post hoc method was applied. In these instances, a Levene’s test was used to confirm equality of variances among the analyzed groups. Differences with *P* < 0.05 were considered statistically significant. Statistical analyses were carried out using SPSS v22.0.0 statistical software (SPSS Inc., Chicago, IL).

## RESULTS

### Genetic ablation of CERK results in decreased levels of C1P in fibroblasts

**Fig. 1.**
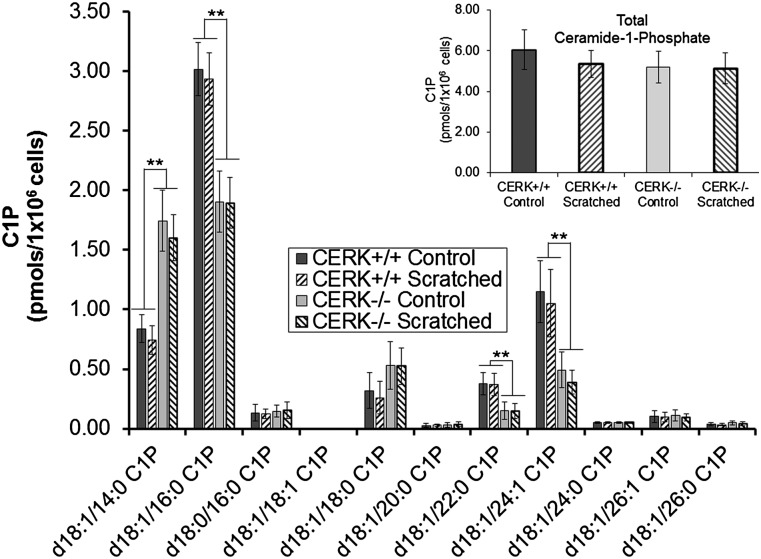
Genetic ablation of CERK translates to a significant loss of specific forms of C1P. Immortalized embryonic fibroblasts (2 × 10^6^) from both CERK^+/+^ and CERK^−/−^ backgrounds were seeded onto 100 mm tissue culture plates and allowed to grow to confluency overnight under standard incubation conditions. Lipids were extracted from these cells, and the lipids species of interest were quantified via HPLC ESI-MS/MS as described in Experimental Procedures. C1P levels are represented in units of picomoles per 1 × 10^6^ cells and are the means of n = 6 of individual experiments ± SD. A one-way ANOVA followed by a Tukey’s post hoc test was used to assess statistical significance (** *P* < 0.01).

In order to ascertain whether CERK ablation has an effect on the intracellular C1P concentrations, we investigated the levels of C1P in MEFs in the presence and absence of mechanical trauma. As anticipated, decreased levels of the major chain length of C1P, d_18:1/16:0_, as well as that of d_18:1/24:1_ (**Fig. 1**), were observed in the CERK^−/−^ cells, confirming the importance of CERK in the synthesis of C1P. Additional decreases were also observed for d_18:1/22:0_ C1P, although this decrease is dependent on culture conditions. Interestingly, d_18:1/14:0_ and d_18:1/18:0_ C1P were found to be elevated in the CERK^−/−^ cells. Neither the wild-type nor the CERK-null fibroblasts demonstrated additional changes to the endogenous C1P content upon induction of mechanical trauma (Fig. 1, inset). These data demonstrate that a significant portion of the d_18:1/16:0_ and d_18:1/24:1_ C1P is CERK derived, but the change in the total levels of C1P is negligible (Fig. 1, inset).

### Genetic ablation of CERK inhibits the ability of fibroblasts to release AA in response to mechanical trauma

**Fig. 2.**
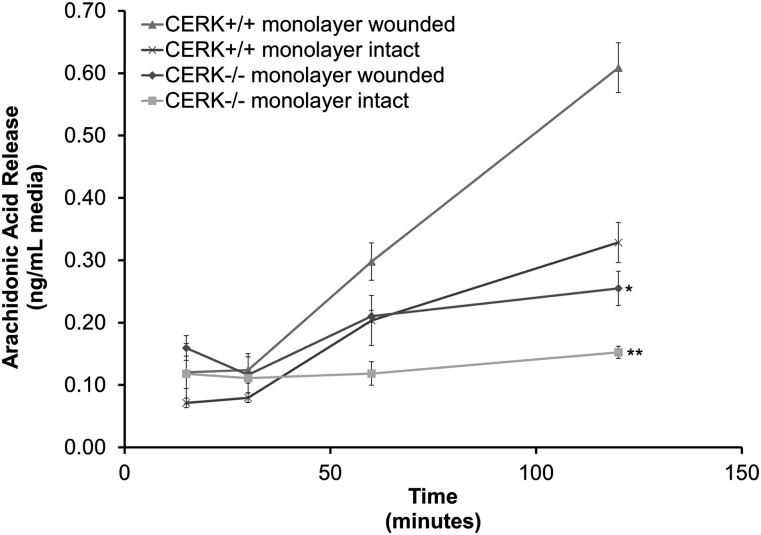
Genetic ablation of CERK and the resultant loss of CERK-derived C1P adversely affects AA release by fibroblasts in response to mechanical trauma. Immortalized embryonic fibroblasts (2 × 10^6^) from both CERK^+/+^ and CERK^−/−^ backgrounds were seeded onto 100 mm tissue culture plates and allowed to grow to confluency overnight under standard incubation conditions in medium containing [^3^H] AA as described in Experimental Procedures. Following the removal of unlabeled AA, the cells were first rested for 2 h in medium containing 2% serum, and the monolayers were either left unharmed as controls or subjected to scratch-induced mechanical trauma as described in Experimental Procedures. The medium was collected at the indicated time points and counted via scintillation. The data are presented as nanograms of AA released per milliliter of medium and are the average of three different experiments ± SD. A one-way ANOVA followed by a Tukey’s post hoc test was used to assess statistical significance of the AA released by the different groups. The analysis revealed significant differences in AA release between wounded CERK^+/+^ and CERK^−/−^ fibroblasts (* *P* < 0.05, ** *P* < 0.01).

Previously, our laboratory demonstrated that C1P produced by CERK was a necessary cofactor in the A23187- and interleukin 1β-induced liberation of cPLA_2_α-dependent AA (16). Thus, CERK-derived C1P may play a role in cPLA_2_α activation in response to mechanical trauma known to induce inflammation. In order to investigate this possibility, we examined the AA release of MEFs in response to scratch-induced mechanical trauma. Over a 4 h period, wild-type MEFs demonstrated increasing basal AA in contrast to CERK^−/−^ MEFs (**Fig. 2**). Upon induction of mechanical trauma by scratching the monolayer, the wild-type MEFs demonstrated a robust increase of liberated AA, while CERK^−/−^ MEFs demonstrated a minor and insignificant increase in AA release (Fig. 2).

The possibility existed that the subdued basal and mechanical trauma-induced AA release by fibroblasts was due to a decrease in the expression of cPLA_2_α. In order to discount this possibility, we compared the expression levels of cPLA_2_α between wild-type and the CERK-null fibroblasts. In agreement with our recent publication (20), there was no significant difference in the expression level of cPLA_2_α between the two genotypes (data not shown). Thus, the genetic loss of CERK adversely affects the AA release in response to mechanical trauma without affecting the expression of cPLA_2_α.

### CERK is required for eicosanoid biosynthesis in response to mechanical insult

**Fig. 3.**
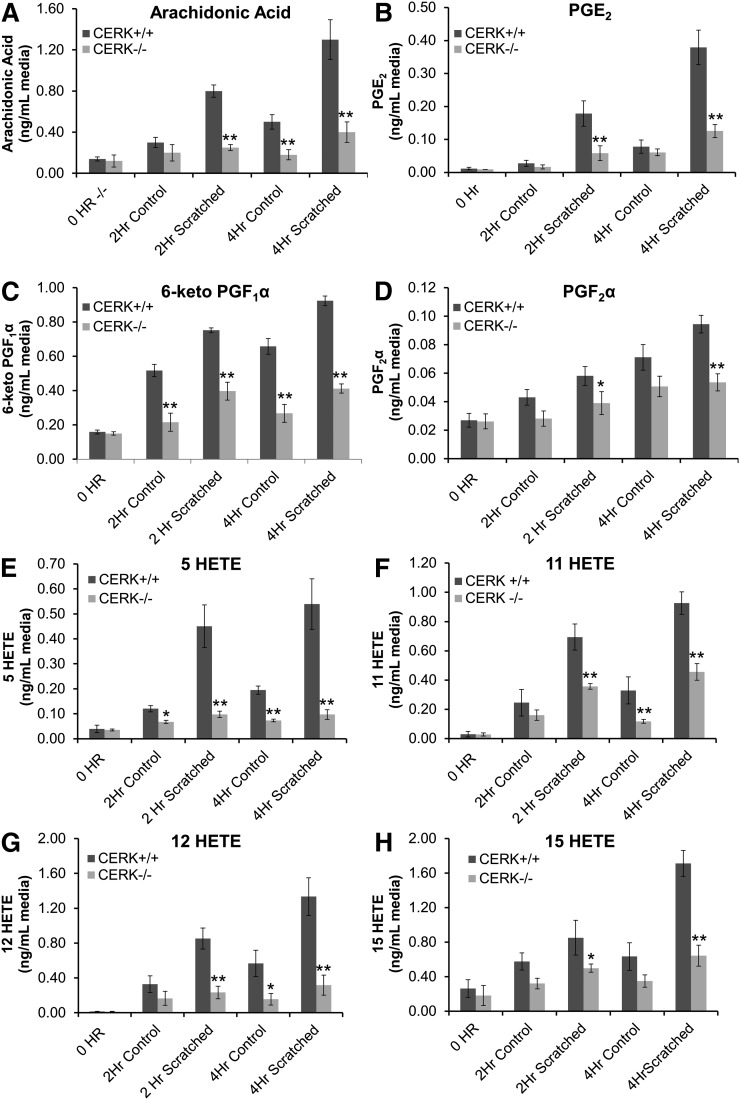
Genetic ablation of CERK and the resultant loss of CERK-derived C1P adversely affects eicosanoid synthesis by fibroblasts in response to mechanical trauma. A–H: Immortalized embryonic fibroblasts (2 × 10^6^) from both CERK^+/+^ and CERK^−/−^ backgrounds were seeded onto 100 mm tissue culture plates and allowed to grow to confluency overnight under standard incubation conditions. Following overnight incubation, the cells were rested for 2 h in medium containing 2% serum, and the monolayers were either left unharmed as controls or subjected to scratch-induced mechanical trauma as described in Experimental Procedures. The eicosanoids secreted into the medium in response to scratch-induced mechanical trauma were quantified at 2 h and 4 h postinjury via HPLC ESI-MS/MS as described in Experimental Procedures. A–H: Quantification of AA, PGE_2_, PGF_2_α, 6-keto PGF_1_α, 5-HETE, 11-HETE, 12-HETE, and 15-HETE, respectively, expressed in units of nanograms lipid per milliliter medium. The data presented are the average of three individual experiments performed on two separate occasions ± SD. Student’s *t*-test was used to assess statistical significance of the difference in scratch-induced eicosanoid release between CERK^+/+^ and CERK^−/−^ fibroblasts (* *P* < 0.05, ** *P* < 0.01).

AA is the precursor of all eicosanoids, and the production of AA is the initial rate-limiting step of eicosanoid synthesis. In order to investigate whether the observed decrease of the release of AA in response to mechanical trauma translated to the level of eicosanoids, we utilized a lipidomic approach using HPLC ESI-MS/MS. Congruent with our findings for the release of steady-state labeled [^3^H] AA (Fig. 2), HPLC ESI-MS/MS analysis of CERK^−/−^ MEFs demonstrated a reduction in both basal and induced AA release compared with CERK^+/+^ MEFs, (**Fig. 3A**) cross-validating the two experimental approaches. Additional lipidomic investigation demonstrated that the decrease in AA release in CERK^−/−^ MEFs translated to reduced synthesis of the prostaglandins PGE_2_ and PGF_2_α, and the prostacyclin metabolite 6-keto PGF_1_α (Fig. 3B–D). Additionally, the substrate limitation was also observed to affect the lipoxygenase products 5-, 11-, 12-, and 15-HETEs (Fig. 3E–H). Taken together, the data indicate that CERK ablation affects both basal and mechanical trauma-induced induction of AA release, which translates into subsequent eicosanoid synthesis. Thus, CERK-derived C1P plays an important and necessary role in the biosynthesis of eicosanoids in response to the wounding of fibroblasts.

### CERK-derived C1P is necessary for the full eicosanoid response of mouse skin to mechanical injury

**Fig. 4.**
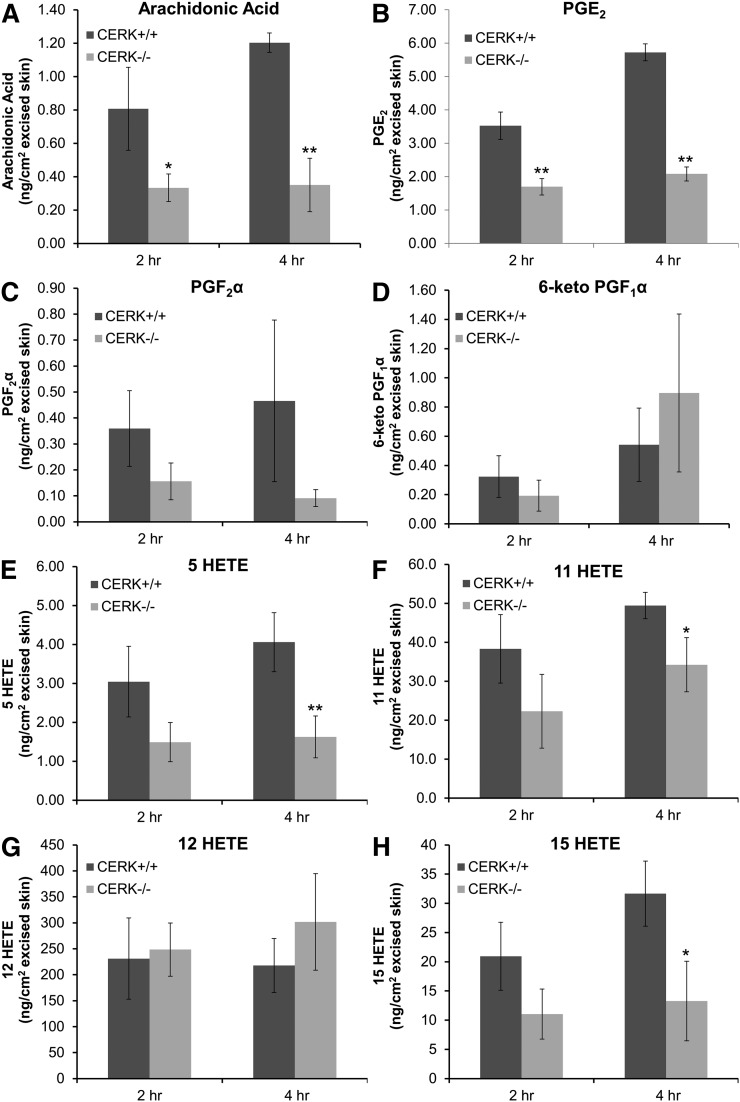
CERK-derived C1P is required for eicosanoid release in response to wounding of the skin. Punch biopsies (10 mm) were obtained from the shaved and depilated dorsum of CERK^+/+^ and CERK ^−/−^ mice according to Experimental Procedures, followed by incubation for 2 h and 4 h under standard incubator conditions in medium containing 2% serum as described in Experimental Procedures. The eicosanoids secreted into the medium from the injured skin were quantified at 2 h and 4 h postbiopsy via HPLC ESI-MS/MS as described in Experimental Procedures. A–H: Quantification of AA, PGE_2_, 6-keto PGF_1_α, PGF_2_α, 5-HETE, 11-HETE, 12-HETE, and 15-HETE, respectively, expressed in units of nanograms lipid per square centimeter excised skin. The data presented are the average of three individual experiments performed on two separate occasions ± SD. Student’s *t*-test was used to assess statistical significance of eicosanoid synthesis by CERK^+/+^ skin biopsy compared with that of CERK^−/−^ (* *P* < 0.05, ** *P* < 0.01).

In order to extend our findings on eicosanoid production to a more complex model, we investigated the synthesis of these lipids by the skin of CERK^−/−^ and CERK^+/+^ mice in response to mechanical injury. Punch biopsies (10 mm full thickness) were obtained from the depilated dorsum of wild-type and CERK-null mice immediately after being euthanized and were transferred to culture medium. The eicosanoids liberated into the medium were quantified over time (2 h and 4 h) via HPLC ESI-MS/MS. Significant reduction was observed in AA, PGE_2_, 5-HETE, 11-HETE, and 15-HETE in the CERK-null mice (**Fig. 4A, B, E, F, H**) compared with the injured skin of their wild-type littermates at 4 h postwounding. In contrast, no significant effects on PGF_2_α, 6-keto PGF_1_α, and 12-HETE were observed between the two groups (Fig. 4C, D, G). Thus, the synthesis of specific eicosanoids in response to injury of the skin of CERK-null mice was severely affected compared with that of the wild-type mice, demonstrating a predominant role for CERK-derived C1P in the initial wound response of the skin.

### CERK-null fibroblasts demonstrate abnormal migration to a wound site

**Fig. 5.**
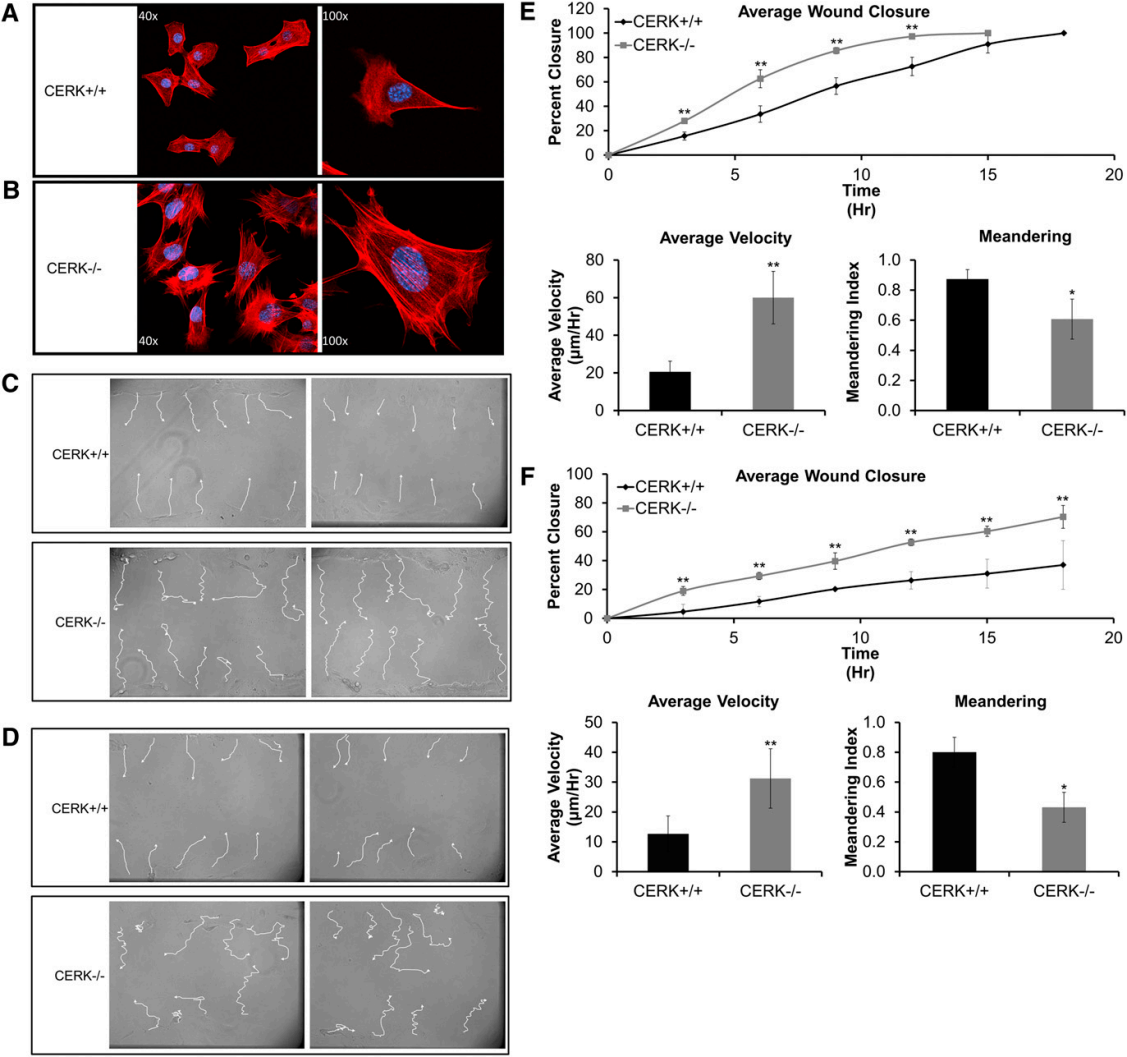
Loss of CERK-derived C1P alters actin distribution and migration of fibroblasts. A–B: CERK^+/+^ and CERK^−/−^ MEFs (0.2 × 10^5^ cells) were seeded overnight onto 22 × 22 mm coverslips in 35 mm plates and stained for actin and DNA as described in Experimental Procedures. C–F: Cells were seeded into 35 mm cell culture dishes and allowed to grow to confluency. Migration was initiated by scratching confluent cells with a p10 pipette tip as described in Experimental Procedures, and the motility was tracked as described in Experimental Procedures. C: Tracings of individual cell migration paths from immortalized CERK^+/+^ and CERK^−/−^ following scratch-induced mechanical trauma. D: Tracings of individual cell migration paths from primary CERK^+/+^ and CERK^−/−^ following scratch-induced mechanical trauma. E: Percent wound closure with time (upper panel) and average velocity of movement and meandering index (lower panels) of immortalized CERK^+/+^ and CERK^−/−^ MEFs. F: Percent wound closure with time (upper panel) and average velocity of movement and meandering index (lower panels) of primary CERK^+/+^ and CERK^−/−^ MEFs.

Multiple studies demonstrate the importance of eicosanoids in fibroblast migration and proliferation (7, 9, 11), an important aspect of the proliferation stage (fibroplasia stage) and the subsequent remodeling phases of wound healing (22). Our findings that MEFs and skin derived from CERK^−/−^ mice have impaired eicosanoid response to wounding raised the possibility of altered migration properties as well. Indeed, the CERK^−/−^ MEFs demonstrated an activated fibroblast phenotype compared with CERK^+/+^ as shown by the presence of more stress fibers by actin staining (**Fig. 5A, B**). In order to directly investigate the role of CERK-derived C1P in migration, a monolayer of MEFs was again subjected to scratch-induced mechanical trauma followed by video microscopic monitoring. Analysis of the individual cell tracks revealed that the CERK-null fibroblasts migrate in a highly random pattern in contrast to their wild-type counterparts (Fig. 5C, D) as measured by the meandering index (Fig. 5E, F, lower right panels). Hence, CERK^−/−^ MEFs have a significant loss in migration polarity. However, the CERK-null fibroblasts demonstrated an overall increase in migration, thereby covering the wound area faster compared with CERK wild-type fibroblasts (Fig. 5E, F, lower left panels). Regardless, these data demonstrate that CERK-derived C1P plays a central role in the proper migration of fibroblasts to a wound site.

### The migratory response to wounding of CERK^−/−^ fibroblasts can be rescued via the addition of exogenous eicosanoids

**Fig. 6.**
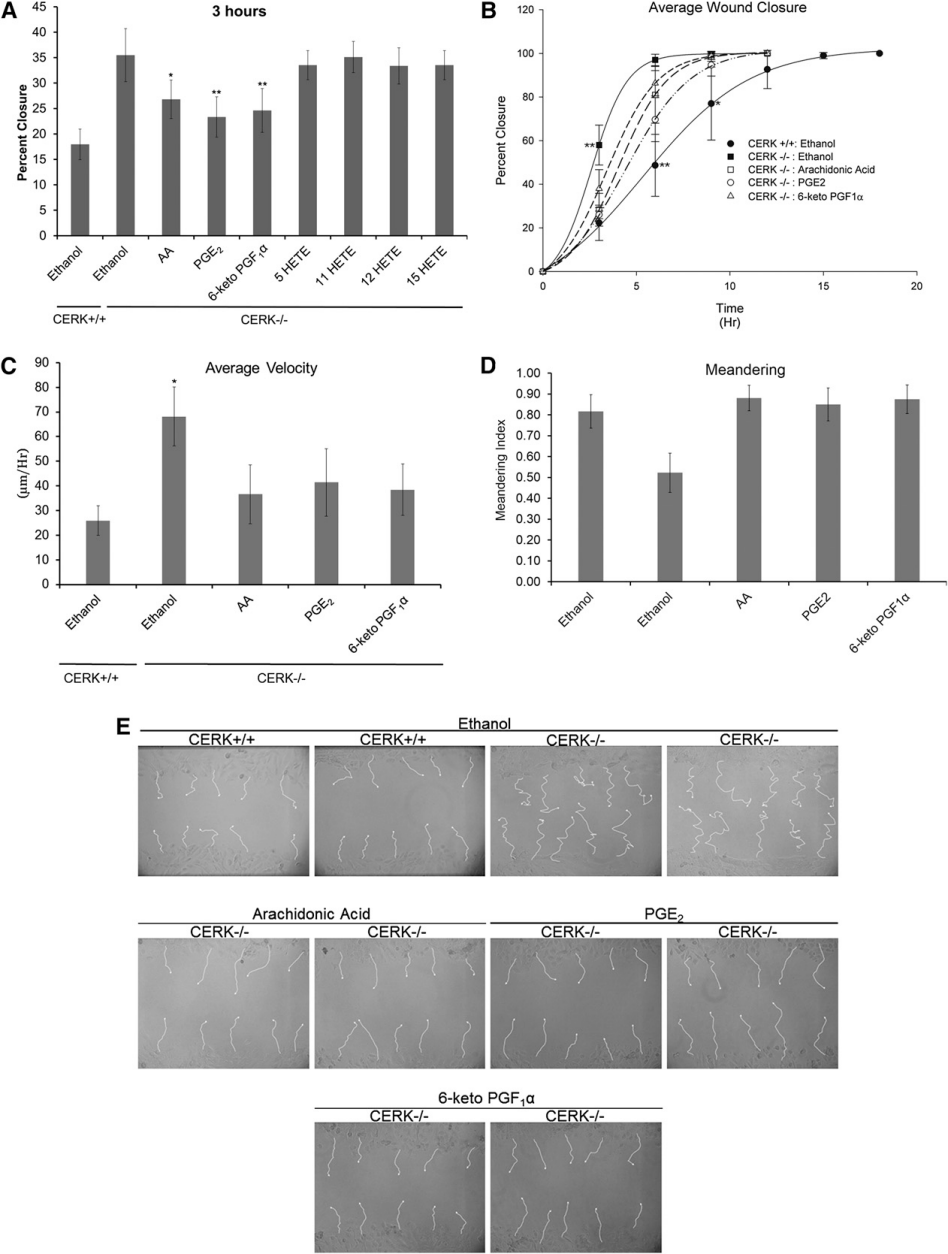
Abnormal migration of fibroblasts due to loss of CERK is partially rescued via the addition of exogenous eicosanoids. A: MEFs (2 × 10^6^) from both CERK^+/+^ and CERK^−/−^ backgrounds were seeded onto 100 mm tissue culture plates and allowed to grow to confluency overnight under standard incubator conditions. Following overnight incubation, the cells were rested for 2 h in medium containing 2% serum. The monolayer was then subjected to scratch-induced mechanical trauma with a 200 μl pipette tip, and the medium was supplemented either with ethanol (vehicle) or the relevant deficient eicosanoids as described in Experimental Procedures. The migration of the fibroblasts to the cleared area was photographically recorded at 3 h postscratch. B-E: Cells were seeded into 35 mm cell culture dishes and allowed to grow to confluency and were rested for 2 h in medium containing 2% serum. The monolayer was then subjected to scratch-induced mechanical trauma cells with a p10 pipette tip, and the medium was supplemented either with ethanol (vehicle) or the relevant deficient eicosanoids as described in Experimental Procedures. Cell motility was tracked as described in Experimental Procedures using video microscopy. B: Percent wound closure with time. C: Average velocity of movement. D: Meandering index. E: Tracings of individual cell migration paths. The data presented are the average of six individual experiments performed on two separate occasions ± SD. A one-way ANOVA followed by a Tukey’s post hoc test was used to assess statistical significance of the migration between CERK^+/+^ and CERK^−/−^ treatment groups (* *P* < 0.05, ** *P* < 0.01).

Our studies have demonstrated that CERK-derived C1P is an important component in the migration of fibroblasts induced by wounding. In order to determine whether CERK-derived C1P regulates fibroblast migration via eicosanoid biosynthesis, we performed “rescue” experiments. Specifically, the eicosanoids downregulated in the CERK^−/−^ cells and skin were exogenously reintroduced into the culture medium to the same concentration experimentally determined for wild-type fibroblasts. The data demonstrate that while the HETEs did not have any effect on the rate of migration of the CERK-null fibroblasts, reintroduction of AA and the COX-derived eicosanoids, PGE_2_ and 6-keto PGF_1_α, induced a significant decrease (i.e., “rescued”) in the migration of CERK^−/−^ MEFs at the early time points of linear migration versus migration time points approaching confluency (**Fig. 6A, B**). To further investigate this phenomenon, wild-type and CERK-null fibroblasts subjected to surface wounding were monitored by video microscopy in the presence and absence AA, PGE_2_, and 6-keto PGF_1_α. These studies again demonstrated a “rescue” of the rate of wound closure by these agents in CERK-null fibroblasts at early time points of linear migration (Fig. 6B). Furthermore, during this early rescue period, both the average velocity and the meandering index of the CERK ^−/−^ were completely “rescued” (Fig. 6C–E). Hence, the enhanced migration and dysregulated polarity of CERK-null fibroblasts is due to the decreased production of COX-2-derived eicosanoids.

### C1P generation in human wounds correlates with the inflammatory and proliferative stages of wound healing

**Fig. 7.**
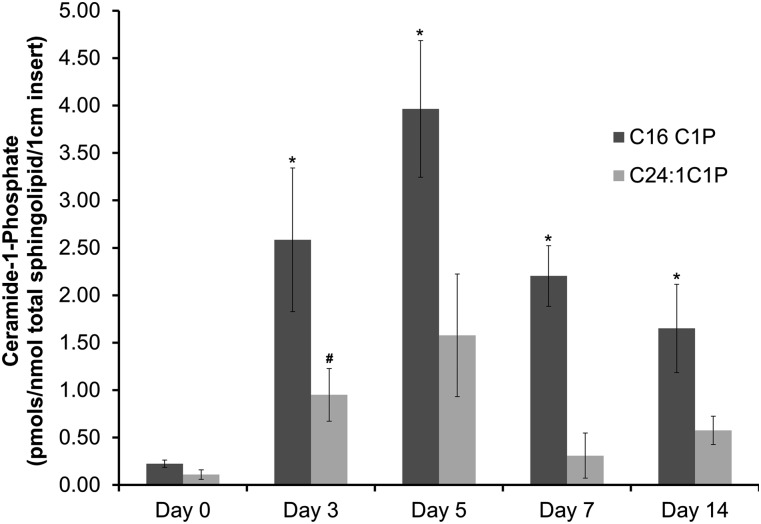
The levels of C1P increase during the early stages of wound healing in human patients. PTFE tubings (3 cm) expanded to 90 μm pore size were implanted subcutaneously in the upper arm using a 14 gauge needle as described in Experimental Procedures, thereby creating an acute wound. A punch biopsy (10 mm) was taken at the same time for comparison (day 0). The tubes were removed on days 3, 5, 7, and 14 allowing cells involved in wound healing to infiltrate. The lipids were extracted from 1 cm length of PTFE tubing and subjected to quantitation via HPLC ESI-MS/MS as described in Experimental Procedures. The data presented are the average from three individuals ± SD. Student’s *t*-test was used to assess statistical significance between C1P levels compared with day 0 (* *P* < 0.05).

In order to extend our findings regarding the importance of C1P in fibroblast migration to wound healing in humans, we investigated changes in the levels of this bioactive lipid in a human acute wound model (**Fig. 7**). The levels of C1P were observed to increase from day 0 through day 5 before decreasing through day 7 to day 14 (Fig. 7). These data indicate a steady increase in C1P through the inflammatory phase reaching a maximum centered on the peak of fibroplasia during the proliferative phase. Hence, there is a tight correlation between the levels of C1P and the migration stage of fibroblast, further implicating an important role for this bioactive lipid in fibroplasia during wound healing.

## DISCUSSION

In this study, our laboratory identified a role for CERK-derived C1P in the wound-healing response of fibroblasts. Specifically, we found that removal of CERK-derived C1P via genetic ablation led to a muted AA release and eicosanoid biosynthesis of fibroblasts in response to scratch-induced mechanical trauma. We also demonstrated that this effect resulted in changes to the rate and the pattern of migration of fibroblasts. Thus, for the first time, we have found a link between the CERK-derived C1P and polarity/migratory process of fibroblasts.

Our study also strengthens the argument for a major role played by CERK-derived C1P in the mediation of important eicosanoid responses. For example, CERK-null mice were reported as mainly aphenotypic (23). Furthermore, genetic ablation of CERK was observed to not affect the pathways of cPLA_2_α activation in certain cell types such as peritoneal macrophages (21).The inference of these findings was that CERK is not central to eicosanoid synthesis. However, in the absence of this CERK-derived pool of C1P (d_18:1/16:0_ and d_18:1/24:1_), a developmental adaptation is observed via the upregulation of the levels of d_18:1/14:0_ and d_18:1/18:0_ C1P through an alternate/unknown route of synthesis as we recently reported (20). Possible alternate mechanisms for anabolism of C1P include an unidentified CERK, a potential mammalian enzyme with sphingomylenase D activity that hydrolyzes choline from sphingomyelin to generate C1P, or an enzyme with acylase activity that generates C1P via acylation of sphingosine-1-phosphate. However, this adaptation is not sufficient to overcome the negative effects of the loss of CERK on eicosanoid biosynthesis in all ex vivo cells. Specifically, fibroblasts have lost a majority of their ability to respond to crucial stimuli such as mechanical trauma. Hence, CERK-derived C1P appears to play a central role in this eicosanoid response, and this role cannot be completely replaced via increases in non-CERK-derived species of C1P. Furthermore, the importance of CERK-derived C1P in the cPLA_2_α-mediated AA response should be evaluated for the specific cell type and the stimulation under investigation and should not be discounted based on the phenotype of a limited number of cell types such as macrophages.

Our data demonstrating impaired ability of fibroblasts to induce a robust eicosanoid response also have implications for the other cells involved in the wound-healing process. For example, numerous reports have demonstrated an interaction between fibroblasts and keratinocytes during wound healing (24). Indeed, fibroblast-generated PGE_2_ is known to influence keratinocyte proliferation (25). Furthermore, when culturing keratinocytes, 3T3 fibroblasts treated with mitomycin c are sometimes used as a feeder line to provide the required growth factors including eicosanoids, indicating the relevance for the keratinocyte-fibroblast interaction (26). Thus, any disruptions in the eicosanoid generation potential of the fibroblasts will most likely affect the keratinocyte proliferation, compromising the epithelialization step of the wound-healing process. Thus, it is likely the CERK is also necessary for the induction of wound epithelialization by keratinocytes, and its loss may lead to nonhealing wounds. Indeed, during the revision of this manuscript, a study was published by Kim et al. (27) demonstrating a relationship between C1P and the migration of multipotent stromal cells and endothelial progenitor cells with similar implications for tissue regeneration. Our results are in accord with their findings in regard to the C1P requirement for fibroblast migration, but differ in that the effect of C1P in fibroblasts is to generate COX-2-derived eicosanoids important for driving migration and cell polarity in an autocrine and paracrine manner. Disease states due to the disruptions in the migration of fibroblasts are not limited to wound healing. There are many other fibroproliferative disorders that are the result of excessive accumulation and influx of fibroblasts and subsequent collagen deposition. These include pulmonary fibrosis, systemic sclerosis, liver cirrhosis, cardiovascular diseases, progressive kidney diseases, and macular degeneration, as well as influencing cancer metastasis and accelerating graft rejection (28). Many of the fibroblasts isolated from fibroproliferative disorders demonstrate an increased migratory capacity (4), proliferation, and increased collagen synthesis that is associated with a diminished ability to produce PGE_2_ (6). Interestingly, we also observed a decrease in C1P toward the end of the fibroplasias stage of wound healing in humans, which would coincide with a decrease in PGE_2_ and induction of collagen synthesis by fibroblasts. Furthermore, the dysregulated migratory and proliferative phenotypes observed in the CERK-null fibroblasts is quite similar to the phenotype of fibroblasts isolated from the fibrotic lung diseases (6). The decrease in PGE_2_ observed in CERK^−/−^ cells is of particular importance as the phenotype of the fibroblasts obtained closely mimicked those isolated from fibroproliferative disorders, suggesting a possible role for CERK in those disorders (e.g., excessive scar and keloid formation following wounding). Indeed, this study also shows that the migration of fibroblasts is slower but very orderly under high PGE_2_ concentration. Thus, while our in vitro wound-healing assay demonstrated faster closure of the simulated wound via enhanced but random migration of CERK-null fibroblasts, in the context of fibroproliferative disorders, loss of CERK functionality may be a detrimental event. Furthermore, uneven migration of these cells can also be expected to result in uneven collagen deposition with consequent effects toward the tensile strength of the healed wound. Hence, cellular C1P content may be an important factor in determining the fibroproliferative phenotype as well as tensile strength of healed wounds, and further studies are warranted to investigate this new paradigm.

While the “rescue” of the migration abnormalities of CERK ^−/−^ with AA and PGE_2_ is consistent with published data and findings, a full “rescue” by 6-keto PGF_1_α was quite unexpected. This eicosanoid is the stable nonenzymatic hydrolysis product of prostacyclin and until now was considered to be biologically inactive. As such, it was included in our assays as a negative control. However, repeated analysis by two different laboratories indicates a role for 6-keto PGF_1_α in the migration of MEFs. Because very little is known regarding the role of eicosanoids in fibroblast migration and general wound healing, these data suggest that future studies should be undertaken to understand what specific role this eicosanoid metabolite plays in this process.

Abnormal migration can be due to deficiencies in one or more factors including eicosanoid signaling. Studies carried out by White et al. (5) demonstrated that lung fibroblast migration is inhibited by PGE_2_ via an E-prostanoid 2 receptor-mediated mechanism that involved increased cAMP production and subsequent phosphatase and tensin homolog (PTEN) activation via decreased tyrosine phosphorylation of PTEN. Indeed, PTEN counteracts the activities of phosphatidylinositol 3-kinase via the dephosphorylation of phosphatidylinositol (3,4,5)-trisphosphate, and the loss of PTEN activity was identified as a cause of enhanced migration of murine fibroblasts (29). The rescue of the migration phenotype of CERK-null fibroblasts by exogenous addition of PGE_2_ may be attributed to the reestablishment of this signaling pathway. Because AA is the direct precursor of PGE_2_, the observed reversal of the enhanced migration phenotype by AA is likely due to the conversion of AA into PGE_2_. However, this proposed mechanism does not explain the rescue by 6-keto PGF_1_α. There is a possibility that this metabolite of prostacyclin is being converted to a more biologically active form, which is responsible for the observed partial rescue. Studies carried out by Wong et al. (30) have demonstrated that 6-keto PGF_1_α can be converted into 6-keto prostaglandin E_1_ with similar properties to prostacyclin. The receptor for prostacyclin, IP also leads to the elevation of cAMP via activation of adenylate cyclase (9) and as such can be expected to inhibit fibroblast migration as described by Kohyama et al. (9) through the same pathway. Thus, the observed abnormalities in the migration of CERK-null fibroblasts that is “rescued” by the exogenous addition of eicosanoids is hypothesized to occur via the adenylate cyclase-PTEN axis of signaling.

In conclusion, we have demonstrated that the loss of CERK results in a decrease of C1P production, which results in significant decreases in many of the eicosanoids produced by fibroblasts. In turn, this decrease in COX-2-derived eicosanoids resulted in the enhanced but random migration of fibroblasts (loss of cell polarity). Our studies in complex wound-healing systems in humans and mice further corroborate this role for CERK-derived C1P in this paradigm. Therefore, this study has implicated the modulation of CERK, and thus C1P, in the enhancement of wound healing as well as ameliorating fibroproliferative disorders.

## Supplementary Material

Supplemental Data
