# Supplementary material for: Ceramide kinase is required for a normal eicosanoid response and the subsequent orderly migration of fibroblasts
Source: J Lipid Res. 2014 Jul;55(7):1298–309. doi: 10.1194/jlr.M048207 (PMC4076082; doi:10.1194/jlr.M048207)
Supplement: Supplemental Data [file supp_55_7_1298__index.html]

Ceramide kinase is required for a normal eicosanoid response and the subsequent orderly migration of fibroblasts — Supplemental Data 

# Ceramide kinase is required for a normal eicosanoid response and the subsequent orderly migration of fibroblasts

## Supplemental Data

Supplemental Table I

**Files in this Data Supplement:**

- Supplemental Data - Supplemental Data
