# Supplementary material for: Ceramide kinase is required for a normal eicosanoid response and the subsequent orderly migration of fibroblasts
Source: J Lipid Res. 2014 Jul;55(7):1298–309. doi: 10.1194/jlr.M048207 (PMC4076082; doi:10.1194/jlr.M048207)
Supplement: Supplemental Data [file supp_M048207_Supplementary_Table_1.docx]

**Supplemental Table 1**

| Species | Precursor Ion (*m/z*) | Product Ion (*m/z*) |
| --- | --- | --- |
| PGF_2_α | 353 | 193 |
| PGF_2_α-d_4_ | 357 | 197 |
| PGE_2_ | 351 | 271 |
| PGE_2_-d_4_ | 355 | 275 |
| PGD_2_ | 351 | 271 |
| PGD_2_-d_4_ | 355 | 275 |
| LTB_4_ | 335 | 195 |
| LTE_4_ | 438 | 235 |
| 6-keto PGF_1_α | 369 | 163 |
| 6-keto PGF_1_α-d_4_ | 373 | 167 |
| 5 HETE | 319 | 115 |
| 5 HETE-d_8_ | 327 | 116 |
| 8 HETE | 319 | 155 |
| 11 HETE | 319 | 167 |
| 12 HETE | 319 | 179 |
| 15 HETE | 319 | 113 |
| 20 HETE | 319 | 145 |
| Arachidonic Acid | 303 | 259 |
| Aracidonic Acid-d_8_ | 311 | 267 |
